# Irritable bowel syndrome and associated mental health problems among Middle East and North African medical students: a multicentric cross-sectional study

**DOI:** 10.1186/s12889-025-25356-7

**Published:** 2025-11-17

**Authors:** Abdelrahman Abdelshafi, Belal Osama, Zeinab G. Abdelhamid, Aboalmagd Hamdallah, Youssef Gouda Youssef, Hamza A. Abdul-Hafez, Sara Sabbagh, Ziad Aljarad, Doaa Mohamed Osman, Doaa Mazen Abdel-Salam

**Affiliations:** 1 Faculty of Medicine, Assiut University, Assiut, Egypt; 2 Faculty of Medicine, Benha University, Benha, Egypt; 3 Department of Public Health and Community Medicine, Faculty of Medicine, Assiut University, Assiut, Egypt; 4 Faculty of Medicine, Al-Azhar University, Damietta, Egypt; 5 Department of Medicine, Faculty of Medicine and Health Sciences, An-Najah National University, Nablus, Palestine; 6 Faculty of medicine, Damascus University, Damascus, Syrian Arab Republic; 7 University of Aleppo, Aleppo, Syrian Arab Republic

**Keywords:** Irritable bowel syndrome, Mental health problems, Associates, And medical students

## Abstract

**Background:**

Irritable bowel syndrome (IBS) is a functional gastrointestinal disorder characterized by abdominal discomfort or pain, accompanied by alterations in bowel habits. Its exact pathophysiology remains unclear, but mental health problems are recognized as a major risk factor. Therefore, this study aimed to identify the prevalence of IBS among medical students and explore its association with various factors, including comorbid mental health issues.

**Methods:**

This multicentric cross-sectional study employed an online self-administered questionnaire, distributed among medical students from 27 faculties of medicine across seven countries in the Middle East and North Africa. Universities were selected using a simple random sampling technique. Possible associates were evaluated using the Generalized Anxiety Disorder Questionnaire, the Patient Health Questionnaire, and the Rome IV Diagnostic Questionnaire for adult IBS.

**Results:**

A total of 8,275 medical students completed the questionnaire, with 40.1% diagnosed with IBS according to the Rome IV criteria. Adjusted logistic regression analysis revealed significant associates of IBS, including female gender (AOR = 1.56, *p* < 0.001), enrollment in the third academic year (AOR = 1.23, *p* = 0.003) or higher (AOR = 1.44, *p* = 0.001), lack of regular exercise (AOR = 1.18, *p* = 0.003), and living alone or on campus (AOR = 1.18, *p* = 0.006). Additionally, a history of food or drug hypersensitivity (AOR = 1.49, *p* < 0.001) and overweight or obesity (AOR = 1.19, *p* = 0.002) were significantly associated with IBS. Medical students experiencing depression (AOR = 2.38, *p* < 0.001) and anxiety (AOR = 1.91, *p* < 0.001) were found to be more susceptible to developing IBS.

**Conclusion:**

IBS is a prevalent health issue among medical students. Nearly 40% of them suffered from IBS. In addition to certain personal and academic factors, increased rates of depression and anxiety among medical students in the Middle East and North Africa are associated with a higher probability of developing IBS.

**Supplementary Information:**

The online version contains supplementary material available at 10.1186/s12889-025-25356-7.

## Background

 Irritable bowel syndrome (IBS) is defined by the Rome IV criteria as recurrent abdominal pain on average at least 1 day/week in the last 3 months, associated with two or more of the following criteria: (i) related to defecation; (ii) associated with a change in frequency of stool; and/or ^(^iii) associated with a change in form (appearance) of stool. Symptoms must have started at least 6 months prior to diagnosis [1, 2], which significantly impact the quality of life with socioeconomic and psychosocial consequences [3]. To diagnose patients with IBS, physicians have to rule out organic diseases such as inflammatory bowel disease or infections. They need to exclude metabolic causes like lactose intolerance, celiac disease, or thyroid disorders. Any structural abnormalities need to be excluded to confirm the diagnosis [4].

IBS is considered a part of functional gastrointestinal disorders, which are a group of gastrointestinal disorders without an underlying organic cause [5, 6]. IBS places a significant burden on healthcare systems. In the United States, annual medical costs for IBS patients have been reported to reach 8 billion dollars. Moreover, due to diagnostic challenges in atypical cases, many patients have undergone unnecessary surgeries [7].

IBS is highly influenced by genetic factors, with 33% of patients having a positive family history [8]. Ethnic factors and psychological factors, such as anxiety, depression, and stress, were reported to be intertwined with IBS [9, 10]. Enteric infections, antibiotic use, and behavioural factors such as unhealthy eating and cigarette smoking, play an important role in the development of IBS [11–13]. According to the published literature, females have a higher prevalence of IBS in comparison to males [8, 14].

A previous study conducted in two Jordanian medical facilities using the Rome III criteria found that 30.9% of medical students had IBS [15]. Another cross-sectional study conducted in King Khalid University in Saudi Arabia, relying on the Rome IV criteria, reported a prevalence of IBS of 10.7% among medical students [16].

Medical students are more likely to develop IBS, with reported prevalence rates ranging from 9.3% to 35.5% [17, 18]. This high prevalence among medical students may be due to special stressful events and the learning environment, limited access to healthy food, and financial restrictions [19]. IBS is much more prevalent among the population who experience stress, depression, and anxiety [19].

Medical students represent a distinct group that undergoes significant cognitive and emotional changes. They have to cope with the heavy study requirements, exams, and the intense competition for jobs. These factors lead to an unhealthy lifestyle, which predisposes them to IBS [12, 13]. However, there is a gap in knowledge specifically addressing the prevalence and impact of IBS among medical students in the Middle East and North Africa (MENA) region. Considering the cultural, dietary, and socioeconomic differences in this region in comparison to other countries, it is important to explore how these factors may influence the occurrence of IBS. This study aimed to fill this gap in knowledge by finding the prevalence of IBS among medical students and exploring its association with different factors, including comorbid mental health problems.

## Methods

### Study design and participants

This multicentric cross-sectional study was conducted across 27 faculties of medicine in seven countries from the Middle East and North Africa, including Egypt, Palestine, Syria, Jordan, Iraq, Libya, and Sudan. Data collection was carried out from March 1 to May 30, 2024, targeting medical students from the first year to the internship years. The present study excluded participants who reported a previous diagnosis of IBS, IBD, gastrointestinal reflux disease, or other functional organic diseases. The authors excluded participants with a previous diagnosis of IBS, as these cases may not have been identified using the Rome IV criteria, which is the standard used in this study. Therefore, they might have been diagnosed using different methods, resulting in heterogeneity in both the diagnostic tools and the diagnosed population. In addition, patients who reported gastrointestinal red flags such as unexplained weight loss, rectal bleeding, anaemia, or a family history of gastrointestinal cancers were not included in this study.

### Sample size calculation

Sample size was calculated using the Epi Info version 7 StatCalc, based on the following assumption: prevalence rate of IBS among medical students (30.9%) [15], level of confidence 99.9%, precision 5%, and design effect 4. The minimum required sample size is 5168 students.

### Sampling technique and data collection approach

A multistage approach was adopted in the present study. First, the faculties of medicine in the seven participating countries were listed, and 27 universities were selected through simple random sampling to ensure geographic and institutional diversity. Within each selected university, a convenience sampling approach was used to invite medical students to participate, primarily via social media platforms.

Respondents completed the questionnaire using Google Forms. They answered the questionnaire anonymously, on their own time, and were not compensated to participate. Data collection was from 27 faculties of medicine in seven countries from the Middle East and North Africa, including Egypt, Palestine, Syria, Jordan, Iraq, Libya and Sudan.

### Data collection tool

The present study assessed IBS prevalence in the Middle East and North Africa. It aimed to estimate the prevalence of anxiety and depression in our targeted population. Sociodemographic and economic factors, as well as other IBS-related associates, were investigated.

A four-part self-reported online questionnaire, available in both Arabic and English languages, was distributed. Each item of the survey assessed a specific aspect associated with our study aims.

The first part included questions inquiring about sociodemographic characteristics, nutritional habits, personal behaviors such as physical exercise, smoking status, and average sleeping hours per night, medical and psychiatric history, as we listed the previous diagnosis of some disorders as exclusion criteria. The students were asked to self-report whether they have food or drug hypersensitivity. Concerning body mass index (BMI), the researchers provide an equation for calculating BMI in the questionnaire to help medical students calculate their BMI. They add the standardized categorization of weight status based on the WHO classification within the questionnaire, with the range of values for each category [20].

The second part was the Generalized Anxiety Disorder seven-item (GAD-7) questionnaire. It has a sensitivity of 89% and a specificity of 82% [21]. This tool is used for screening anxiety disorder and measuring its severity [22]. It included questions about nervousness, inability to stop worrying, excessive worry, restlessness, difficulty relaxing, easy irritation, and fear of something awful happening. Each of the seven items was measured on a three-point scale according to the answer, which ranges from (Not at all) to (Nearly every day) [22].

In the third part, the researchers intended to investigate depression symptoms among the targeted population. For that purpose, the researchers employed the Patient Health Questionnaire (PHQ-9), which consists of nine questions that estimate the frequency of depressive symptoms over the past two weeks, using the same scoring scale as the previous part [23]. GAD-7 and PHQ-9 have been validated in the Arabic language [24].

**Fig. 1:**
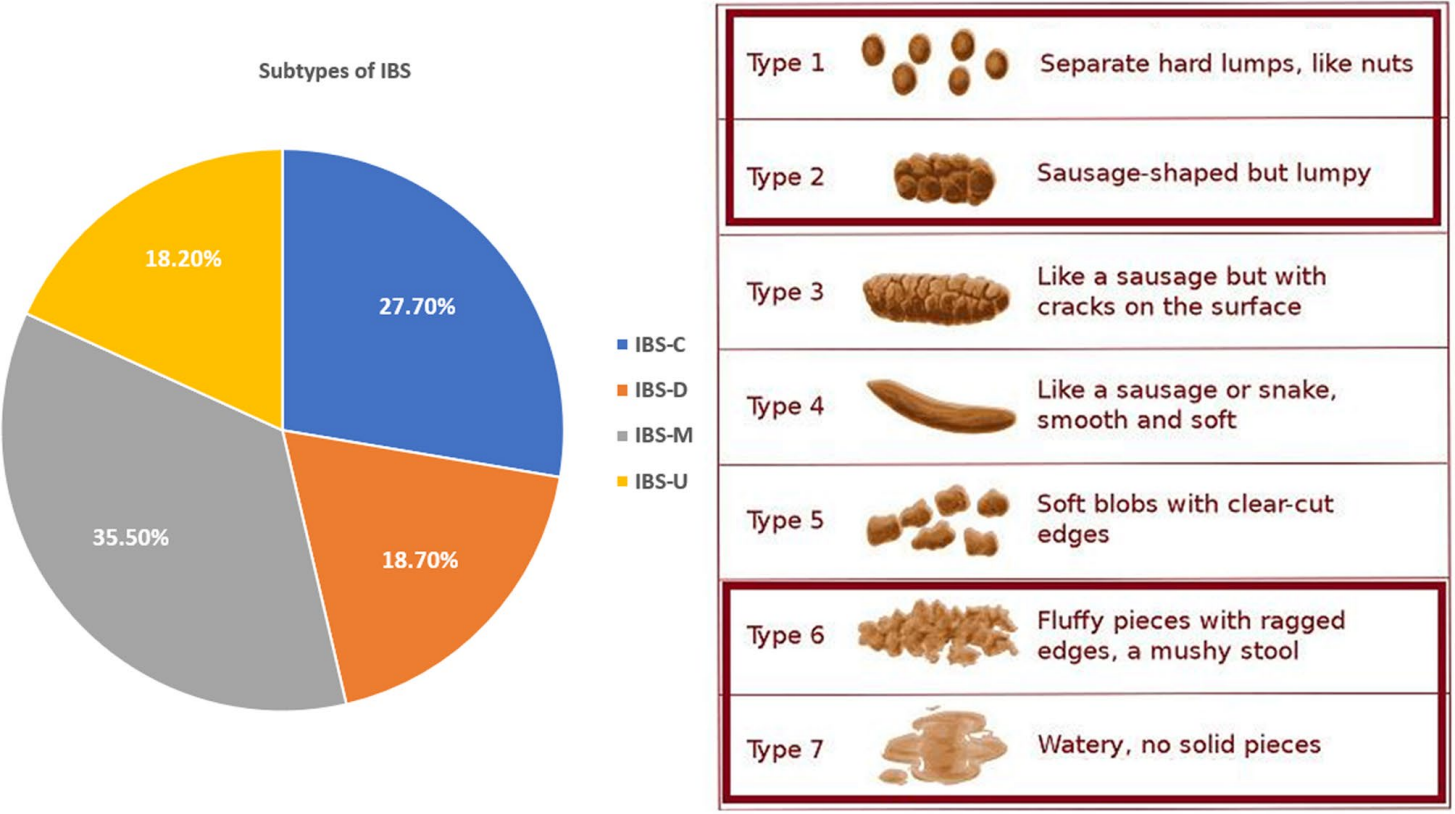
The distribution on different types of IBS among studied medical students according to Rome IV criteria. IBS-C refers to IBS with predominant constipation, IBS-D refers to IBS with predominant diarrhea, IBS-M refers to IBS with mixed bowel habits, and IBS-U refers to IBS unclassified

The fourth part was the Rome IV diagnostic questionnaire for adults with IBS, which includes specific criteria that must be met to set up a diagnosis of IBS, provided that symptoms have appeared not less than six months before diagnosis [25]. These include recurrent abdominal pain at least one day a week in the last three months, associated with defecation, altered stool frequency, and abnormal stool appearance, or at least two of the mentioned [25]. An illustration was used to determine the consistency of stools (Fig. 1). The Rome IV diagnostic criteria have shown adequate sensitivity and excellent specificity [26]. The authors assessed the internal consistency of the Rome IV diagnostic criteria to diagnose IBS among the studied population. The scale has a high consistency value with a Cronbach’s Alpha value of 0.840.

The prevalence of each IBS subtype is determined according to Rome IV criteria which divides the IBS into four subtypes using Bristol Stool Scale for feces characterization: IBS with predominant constipation (IBS-C): More than one fourth (25%) of bowel movements with Bristol stool form types 1 or 2 and less than one-fourth (25%) of bowel movements with Bristol stool form types 6 or 7. IBS with predominant diarrhea (IBS-D): more than one-fourth (25%) of bowel movements with Bristol stool form types 6 or 7 and less than one-fourth (25%) of bowel movements with Bristol stool form types 1 or 2. IBS with mixed bowel habits (IBS-M): more than one-fourth (25%) of bowel movements with Bristol stool form types 1 or 2, and more than one-fourth (25%) of bowel movements with Bristol stool form types 6 or 7. IBS unclassified (IBS-U): Patients who meet diagnostic criteria for IBS but whose bowel habits cannot be accurately categorized into 1 of the 3 groups above should be categorized as having IBS unclassified [27] (Fig. 1). The questionnaire was attached as a supplementary file.

Before the collection of data, the researchers carried out a 300-participant pilot study to appraise the time needed to complete the questionnaire, as well as to ensure that it was clear, practicable, and feasible.

#### Statistical analysis

The data were analyzed using IBM SPSS Statistics for Windows, Version 26 (Released 2019; IBM Corp., Armonk, New York, United States). Each categorical variable was represented as frequencies and percentages. The numerical variable was presented as the mean and standard deviation. Unadjusted and adjusted logistic regression models were applied to identify the associates of IBS. In the regression models, the outcome variable was IBS status as a binary variable (IBS positive = 1, IBS negative = 0). The significant variables that yielded in the unadjusted models were entered into the adjusted model. The explanatory variables entered in the models were qualitative variables, except age, which was entered as a quantitative variable.

The absence of multicollinearity was tested through the correlation matrix for all the entered variables in the adjusted logistic regression model. The researchers ensured that the correlation between any of the entered variables did not exceed 0.7. Results of the regression models were presented using the Odds ratio and 95% confidence intervals. P-values less than 0.05 were taken as a cutoff level for considering the significance in unadjusted and adjusted logistic regression models.

## Results

The present study showed that 40.1% (*n* = 3317) of the medical participants studied had IBS. Table 1 displays the distribution of IBS by personal characteristics among the studied medical students. The mean age of the students was 21.89 ± 2.2, which was higher among IBS positive students. IBS was higher among females in comparison to males (45.4% versus 30.4%, respectively). Higher proportions of IBS were shown among interns (44.7%), third year (41.1%), and fourth year medical students (40.4%). Furthermore, IBS was higher among tobacco nonsmokers. Concerning parents’ marital status, IBS was higher among medical students whose parents were not living together or divorced (47.4%). Students who lived alone reported higher IBS symptoms (45.2%).

**Table 1:** Distribution of IBS by personal characteristics among studied medical students in the middle East and North African regions

Variable	Total (*n* = 8275)	IBS Positive (*n* = 3317)	IBS Negative (*n* = 4958)
	No. (%)	No. (%)	No. (%)
Age (Mean ± SD)	21.89 ± 2.2	21.96 ± 2.199	21.85 ± 2.194
Gender			
Male	2936 (35.5)	893 (30.4)	2043 (69.6)
Female	5339 (64.5)	2424 (45.4)	2915 (54.6)
Academic year			
First year	917 (11.1)	343 (37.4)	574 (62.6)
Second year	1220 (14.7)	475 (38.9)	745 (61.1)
Third year	1587 (19.2)	652 (41.1)	935 (58.9)
Fourth year	1193 (14.4)	482 (40.4)	711 (59.6)
Fifth year	1755 (21.2)	677 (38.6)	1078 (61.4)
Sixth year	505 (6.1)	197 (39.0)	308 (61.0)
Interns	1098 (13.3)	491 (44.7)	607 (55.3)
Tobacco smoking			
Yes	963 (11.6)	341 (35.4)	622 (64.6)
No	7312 (88.4)	2976 (40.7)	4336 (59.3)
History of chronic health problems			
Yes	1522 (18.4)	602 (39.6)	920 (60.4)
No	6753 (81.6)	2715 (40.2)	4038 (59.8)
Marital status			
Single	7691 (92.9)	3082 (40.1)	4609 (59.9)
In relation	584 (7.1)	235 (40.2)	349 (59.8)
Parents’ marital status			
Living together	6947 (84.0)	2755 (39.7)	4192 (60.3)
Not living together/divorced	508 (6.1)	241(47.4)	267 (52.6)
One or both parents dead	820 (9.9)	321(39.1)	499(60.9)
Living situation			
With family	6516 (78.7)	2572(39.5)	3944(60.5)
Alone	637 (7.7)	288(45.2)	349(54.8)
Campus	1122 (13.6)	457(40.7)	665(59.3)

IBS proportion was higher among overweight (44.8%) and obese medical students (48.0%). A higher prevalence of IBS was found among individuals consuming junk food (41.0%), with a history of food or drug sensitivity (51.2%), and those who exercised irregularly (42.8%).Regarding mental health problems, IBS was higher among students with depression (55.2%), and anxiety symptoms (49.6%) (Table 2).

**Table 2:** Distribution of IBS by different health behaviors, obesity, depression, and anxiety status among studied medical students in the middle East and North African regions

Variable	Total (*n* = 8275)	IBS Positive (*n* = 3317)	IBS Negative (*n* = 4958)
	No. (%)	No. (%)	No. (%)
Weight categories based on BMI classification			
Underweight	1134 (13.7)	498(43.9)	636(56.1)
Normal	5151(62.2)	1916(37.2)	3235(62.8)
Overweight	1644 (19.9)	737(44.8)	907(55.2)
Obese	346 (4.2)	166(48.0)	180(52.0)
Eating junk food			
Yes	2211 (26.7)	2488(41.0)	3576(59.0%)
No	6064 (73.3)	829(37.5)	1382(62.5)
History of food or drug hypersensitivity			
Yes	1043 (12.6)	534(51.2)	509(48.8)
No	7232 (87.4)	2783(38.5)	4449((61.5)
Exercise regularly			
Yes	2360 (28.5)	783(33.2)	1577(66.8)
No	5915 (71.5)	2534(42.8)	3381(57.2)
Sleeping hours			
< 8 h/day	4221 (51.0)	1719(40.7)	2502(59.3)
≥ 8 h/day	4054 (49.0)	1598(39.4)	2456(60.6)
Depressio			
Yes	3843(46.4)	2123 (55.2)	1720 (44.8)
No	4432(53.6)	1194 (26.9)	3238 (73.1)
Anxiety			
Yes	5196(62.8)	2576 (49.6)	2620 (50.4)
No	3097 (37.2)	741 (24.1)	2338 (75.9)

*BMI* Refers to body mass index

Table 3 shows the adjusted and unadjusted logistic regression models for the prediction of IBS among medical students studied. In the adjusted model, females and students with a history of food or drug hypersensitivity were one and half times more liable for developing IBS compared to males and those without history of such sensitivity (AOR = 1.55; *p* = < 0.001, AOR = 1.49; *p* = < 0.001 respectively). Significant higher odds ratios were shown among third to fourth academic year students (AOR = 1.23; *p* = 0.003), fifth academic year students and above (AOR = 1.44; *p* = 0.001) and students without regular exercise practicing (AOR = 1.17; *p* = 0.003). Students living alone or at campus (AOR = 1.17; *p* = 0.006), and overweight/obese students (AOR = 1.18; *p* = 0.002) were more likely to have IBS. Furthermore, students with anxiety (AOR = 1.91; *p* = < 0.001) or depression (AOR = 2.38; *p* = < 0.001) had nearly twice the risk of developing IBS.

**Table 3:** Adjusted and unadjusted logistic regression models for the prediction of IBS among studied medical students in the middle East and North African regions

Variable	Unadjusted Models		Adjusted model	
	OR (95% CI)	*p*-value	AOR (95% CI)	*p*-value
Age in years	1.022 (1.001–1.042)	0.036*	0.994(0.963–1.026)	0.697
Gender				
Male	Reference group		Reference group	
Female	1.902(1.729–2.093)	< 0.001*	1.559 (1.402–1.734)	< 0.001*
Academic year				
First/Second	Reference group		Reference group	
Third to Fourth	1.072(0.965–1.191)	0.197	1.233(1.072–1.418)	0.003*
Fifth or above	1.212(1.063–1.383)	0.004*	1.440(1.170–1.773)	0.001*
Smoking				
No	Reference group		Reference group	
Yes	0.799 (0.694–0.919)	0.002*	0.917(0.785–1.071)	0.274
Regular exercise				
Yes	Reference group		Reference group	
No	0.662(0.599–0.732)	< 0.001*	1.176(1.055–1.310)	0.003*
Living place				
With family	Reference group			
Alone/In campus	1.127(1.012–1.254)	0.029*	1.176(1.047–1.321)	0.006*
Sleeping hours				
≥ 8 h	Reference group			
Less than 8 h	1.056(0.967–1.153)	0.225		
Having a chronic disease				
No	Reference group			
Yes	0.973(0.869–1.090)	0.640		
History of food/drug hypersensitivity				
No	Reference group		Reference group	
Yes	1.677(1.472–1.911)	< 0.001*	1.492(1.299–1.715)	< 0.001*
Junk food				
No	Reference group		Reference group	
Yes	1.160(1.049–1.282)	0.004*	1.031(0.926–1.149)	0.577
Weight categories based on BMI				
Underweight/Normal weight	Reference group		Reference group	
Overweight/Obese	1.332(1.203–1.475)	< 0.001*	1.188(1.065–1.325)	0.002*
Family status				
Living together	Reference group			
One or both parents are dead/not living together/divorced	1.116 (0.991–1.257)	0.070		
Single status				
Single	Reference group			
Not single	0.993(0.837–1.179)	0.937		
Anxiety				
No	Reference group		Reference group	
Yes	3.102(2.810–3.425)	< 0.001*	1.913(1.710–2.140)	< 0.001*
Depression				
No	Reference group		Reference group	
Yes	3.347(3.053–3.670)	< 0.001*	2.382(2.146–2.644)	< 0.001*

*BMI* Refers to body mass index

**Fig. 2:**
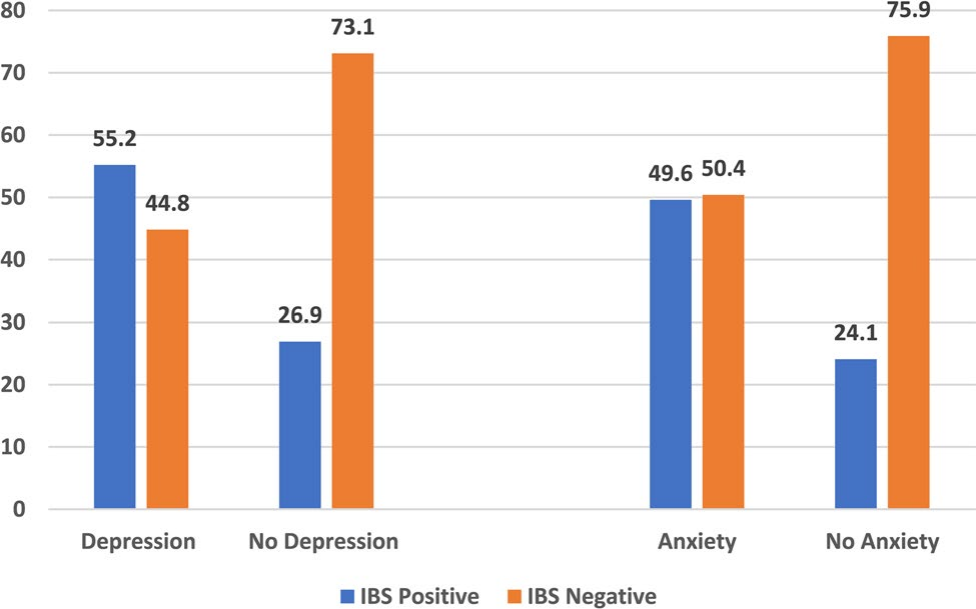
Distribution of IBS among studied medical students wita depression and anxiety status. Data was presented in percentage form

Figure 2 revealed that a higher proportion of IBS was detected among depressed medical students (55.2%) compared to non-depressed (26.9%). Similarly, a higher proportion of IBS was detected among anxious medical students (49.6%) compared to those without anxiety (24.1%).

## Discussion

The present study revealed that 40% of medical students have IBS. IBS was significantly associated with females, students in their third year or higher, an elevated score of depression, and increased anxiety scores. Furthermore, it was linked with a high BMI, individuals living alone, absence of physical activity, and a background of food or drug hypersensitivity.

The present study reveals a higher prevalence of IBS among medical students, as assessed by the Rome IV criteria, compared to previous research. For instance, IBS prevalence was reported as 26.21% at Ibb University, Yemen [28], 22.88% across various universities in Bangladesh [29], 16.9% at the Peruvian University in Peru [30], and 30.9% in northern Jordan universities [16]. Other studies in Egypt show 27.5% at Tanta and Kafr El-Sheikh Universities [31], while it was 31.7% at Ain Shams University [32].

In Saudi Arabia, IBS prevalence was lower at King Abdulaziz University [33], while it reached a higher prevalence than the present study at King Faisal University [34]. A recent Lebanese study reported a higher prevalence of IBS among medical students [35]. The variations in the results may be caused by several factors, such as demographics, socio-cultural variance, and differences in methodological approaches, such as data collection and scoring. Results of the Rome Foundation Global Study reported a significant difference between Rome III and IV Criteria scores in diagnoses of IBS, Rome IV Criteria diagnosing a lower prevalence than Rome III Criteria [36].

The present study revealed a significant association between IBS and mental health problems, including depression and anxiety in agreement with other studies. A study conducted in Malaysia revealed that depression is associated with a 4.7-fold increase in the risk of IBS [37]. Another study done in Malaysia showed a higher prevalence rate of depression among IBS students [38]. Similarly, a Saudi study reported that anxiety was the second correlate of IBS among medical students [33]. A study conducted in France estimated the increase in risk of IBS at a rate of 1.1 with depression and 1.2 with anxiety [39]. A previous meta-analysis including medical students reported that depression and anxiety were associated with over twice the odds of IBS [40]. In Japan, Okami et al. explored a significantly higher level of anxiety and depression among IBS students [41].

The role of food or drug hypersensitivity in IBS isn’t fully understood. Many people have IBS symptoms when they consume certain foods or medicines [42]. The present study revealed that medical students with IBS reported higher levels of food or drug hypersensitivity, in agreement with other studies [42, 43].

In terms of the relation between age and IBS, a small difference in mean age between groups was statistically significant, which may be attributed to the large sample size. However, it is unlikely to reflect a clinically meaningful distinction.

The current study, along with others, has shown a higher occurrence of IBS among females compared to males. The present study findings align with results in previous studies [44, 45]. It is thought that female hormones, particularly during menstruation, impact gastrointestinal function, resulting in a higher occurrence of IBS symptoms in women [46].

The current study showed that students without regular physical activity were at a higher probability of developing IBS consistent with other studies. A study from Saudi Arabia found that IBS prevalence was significantly higher among students who didn’t engage in regular physical activity compared to those who did [33]. Similarly, a study on medical students in Japan discovered that males with IBS reported lower levels of physical exercise compared to their non-IBS students [41]. The same results were observed in Indian medical students, where a significant relation was found between insufficient physical activity and IBS prevalence [47].

Furthermore, research from Korea and China revealed that low levels of exercise were associated with an increased likelihood of developing IBS among university students [48, 49]. Consistent with these findings, regular exercise is not associated with IBS, as most of the students with IBS in one study did not engage in regular physical exercise [32]. Many students face barriers to physical activity due to busy schedules and chronic abdominal pain associated with IBS, so targeted interventions promoting physical activity are needed to reduce IBS risk among student populations [50].

The relationship between sleep disturbances and IBS has produced mixed findings across different populations. The present study showed no significant difference between sleeping hours and the prevalence of IBS. For instance, studies among Egyptian and Malaysian students indicated no significant associations between sleep hours and IBS [31, 37]. However, other research highlights sleep disturbances as a predictor for IBS. A Saudi medical student who slept less than eight hours per day was found to have a higher likelihood of exhibiting IBS symptoms [33]. Students with insomnia reported higher rates of IBS [51]. Japanese nursing and medical students with IBS tended to have later bedtimes [41].

This study showed that smoking was not a significant predictor of IBS in the adjusted regression model coinciding with a study conducted in Jeddah, Saudi Arabia which showed no significant difference between smoking and the prevalence of IBS [33]. In contrast, a study conducted in India revealed a weak association between smoking and a higher incidence of IBS, indicating that regional and cultural factors may influence these findings [47]. Furthermore, a study from Saudi Arabia demonstrated a strong link between smoking and IBS among medical students; these results may be due to the small number of smokers in the study sample [52].

The prevalence of IBS was significantly associated with the academic year of students, peaking among interns and lowest among first-year students. Furthermore, the academic year represented a significant associate for developing IBS in our regression analysis, especially among students in their third year or higher. A study of medical students and interns in Jeddah, Saudi Arabia, found that IBS was higher in advanced academic years, likely due to clinical workload [33]. A study conducted in Bangladesh revealed that medical students in the second study phase were more liable to IBS in comparison to first study phase [29].

The findings of this study indicated that overweight and obese students had a significantly higher prevalence of IBS compared to those in other BMI categories. Overweight and obesity showed an association with IBS in our regression analysis. A study conducted at King Saud University in Saudi Arabia demonstrated that these factors contributed to IBS symptoms [51]. Similarly, a study in Japan showed that students with IBS tended to consume more processed foods and fewer fresh foods [41]. Previous studies indicate that fatty foods, alcohol, caffeine, and lactose (in lactose-intolerant individuals) can exacerbate IBS symptoms [47]. An important finding of this study was the association between living alone or on campus and the greater susceptibility to IBS. After adjusting for other variables that could affect the results, it was shown that students living alone or on campus had 1.17 times higher chances of having IBS compared to those who lived with their families. This is consistent with other studies that have established a relation between living alone or on campus and various health problems, including gastrointestinal disorders [5, 25].

### Strengths and limitations

The present study provides valuable information on IBS and its associates among medical students in the Middle East and North Africa. It has many methodological strengths, including the recruitment of a large number of medical students from different countries, and applying standardized tools for assessment of weight status, IBS, anxiety, and depression disorders. However, it still has certain limitations. The study applied convenience sampling technique and recruited participants through online self-administered questionnaires, which may introduce selection bias. Medical students who frequently engage in online platforms and social media are more likely to participate, potentially skewing the sample toward a certain subgroup while excluding those less active online.

Data collection depended entirely on self-reported responses, which can lead to misclassification as participants might misunderstand questions or inaccurately report their symptoms. Furthermore, the stigma associated with some variables such as depression and anxiety, may lead to social desirability bias because some respondents may modify their answers to align with socially acceptable norms rather than provide an objective assessment.

Regarding the evaluation of regular exercise and sleep quality in the current study, there is a potential for interpretation bias, as these variables were assessed through direct self-report questions rather than standardized methods. The present study applied a cross-sectional study design. The applied study design does not permit a clear distinction between cause and effect. Finally, although data collection methods have been standardized, cultural, socioeconomic, and environmental factors remain significant confounders, affecting IBS prevalence and its associates uniquely in each country. These variations make it challenging to account for all potential confounding variables across different regions.

## Conclusion

IBS is a prevalent problem among Middle Eastern and North African medical students, where 40% of them suffering from IBS. IBS is related to some demographic characteristics, increased BMI, low physical activity, and mental disorders such as anxiety and depression. The present study highlights the burden of some mental health problems among medical students. Screening and proper management of these problems may mitigate the burden of IBS among medical students. Physical exercise should be encouraged with the provision of subsidized services within the University campus.

## Supplementary Information

Supplementary Material 1.

Supplementary Material 2.

## Data Availability

The data of the current study is available from the corresponding author upon request.
