# Supplementary material for: Irritable bowel syndrome and associated mental health problems among Middle East and North African medical students: a multicentric cross-sectional study
Source: BMC Public Health. 2025 Nov 17;25:3968. doi: 10.1186/s12889-025-25356-7 (PMC12621377; doi:10.1186/s12889-025-25356-7)
Supplement: Supplementary file 2 — Supplementary Material 2. [file 12889_2025_25356_MOESM2_ESM.docx]

**The Prevalence of Irritable Bowel Syndrome among Medical Students in the Middle East and North Africa**

You are invited to participate in a research study on The Prevalence of Irritable Bowel Syndrome among Medical Students in the Middle East And North Africa, All information you provide will be kept strictly confidential and there will be no questions that reveal your identity. The survey will take no more than five minutes to complete. The information you provide us with is very valuable to us and will be kept securely in digital form. Please, if you like to participate, agree to fill out the questionnaire.

1.1. Do you consent to participate in this Study?

- Yes I agree
- No I don’t

**University**

2.1. Kindly choose your university

- Tanta University
- Mansoura University
- Benha University
- Assiut University
- Menofia University
- Zagazig University
- Alexandria University
- Kafr El Sheikh University
- Fayoum University
- Helwan University
- Beni-Suef University
- South Valley University
- Al-Azhar University
- Cairo University
- Suez University
- An-Najah National University
- Al-Quds University
- Hebron University
- Arab American University
- Palestine Polytechnic University
- Damascus University
- Al Andalus University for Medical Sciences
- Hama University
- Tishreen University
- Al-Baath University
- University of Aleppo
- Al-Sham Private University
- Syrian Private University
- Tripoli University
- Tobruk University
- University of Gharyan
- University of Benghazi
- Yarmouk University
- Jordan University of Science and Technology
- Omdurman Islamic University
- Karary University
- University of Khartoum
- Bahria University
- Al Neelain University
- Alzaiem Alazhari University
- Sudan University of Science & Technology
- University of Sharjah
- University of Oran 1 Ahmed Ben Bella
- University Abu Bekr Belkaid
- Nahrain University

**Socio-demographic characteristics**

3.1. Age

__________

3.2. Sex

- Female
- Male

3.3. Academic Year

- First Grade
- Second Grade
- Third Grade
- Fourth Grade
- Fifth Grade
- Sixth Grade
- Seventh Grade
- Intern

3.4. Marital Status

- Single
- Married
- Engaged
- Widow
- Divorced
- Separated

3.5. BMI

BMI= Weight (in Kg)/Height^2(in M)

- underweight—-18>
- thin for height —- 18.5>
- healthy weight — 24.9 - 18.6
- Overweight — 29.9 - 25
- Obesity — 30<

3.6. Do you exercise regularly?

- Yes
- No

3.7. On average, how many hours do you sleep per night?

- <8 h/day
- ≥8 h/day

3.8. Do you currently smoke cigarettes or use any other tobacco products?

- Yes
- No

3.9. How is your evaluation rate?

- By percentage
- By GPA

3.10. If Percentage

What is your last evaluation?

- <60%
- 60-69%
- 70-79%
- 80-89%
- 90-100%

3.11. If GPA

What is your last GPA?

- <2
- 2-3
- 3-4

3.12. On average. how many servings of junk food do you have per week?

- Never
- 1-3
- ≥4

3.13. With whom do you live?

- With family
- Alone
- Campus

3.14. What about your parents' relationship? (marital status)

- Living together
- Not living together
- Divorced
- One of my parents is dead
- Both my parents are dead

3.15. Does your income meet your financial goals?

- Enough and exceeds
- Enough Only
- Not Enough

**Medical History**

4.1. Have you ever been diagnosed with irritable bowel syndrome (IBS) by a doctor?

- Yes
- No
- Unknown

4.2. Do you have a history of Chronic health problems?

- Yes
- No

4.3. Which of these medications do you currently use?

- Antidepressants
- Antibiotics
- Anti-inflammatory drugs
- Not using any drugs
- Other.

4.4. Do you have a history of Food or Medical hypersensitivity?

- Yes
- No

4.5. Have you ever been diagnosed with Ulcerative colitis (UC)?

- Yes
- No

4.6. Have you ever been diagnosed with Crohn's disease?

- Yes
- No

4.7. Which of the following diseases have you ever been diagnosed with?

- Gastroesophageal Reflux Disease
- Functional dyspepsia (indigestion)
- Functional nausea and vomiting
- Functional abdominal pain
- Functional constipation
- Functional diarrhea
- Fecal incontinence
- Helicobacter pylori (H. pylori) infection
- Microscopic Colitis
- Inflammatory Bowel Disease (IBD)
- I have never been diagnosed with any

4.8 Do you complain one of the following symptoms?

- Unexplained weight loss.
- Rectal bleeding
- Anemia
- None

4.9 Do you have family history of gastrointestinal cancers

- Yes
- No

4.10. Have you ever been diagnosed with Anxiety?

- Yes
- No

4.11. Have you ever been diagnosed with Depression?

- Yes
- No

**GAD-7 Anxiety**

Over the last two weeks, how often have you been bothered by the following problem:

- 1. Feeling nervous, anxious, or on edge
     - Not at all
     - Several days
     - More than half the days
     - Nearly every day
  2. Not being able to stop or control worrying
     - Not at all
     - Several days
     - More than half the days
     - Nearly every day
  3. Worrying too much about different things
     - Not at all
     - Several days
     - More than half the days
     - Nearly every day
  4. Trouble relaxing
     - Not at all
     - Several days
     - More than half the days
     - Nearly every day
  5. Being so restless that it is hard to sit still
     - Not at all
     - Several days
     - More than half the days
     - Nearly every day
  6. Becoming easily annoyed or irritable
     - Not at all
     - Several days
     - More than half the days
     - Nearly every day

5,7 Feeling afraid, as if something awful might happen

- - - Not at all
    - Several days
    - More than half the days
    - Nearly every day

**PHQ-9 (Patient Health Questionnaire-9)**

How often have you been bothered by the following over the past 2 weeks:

- 1. Little interest or pleasure in doing things like housework or watching TV?
- Not at all
- Several days
- More than half the days
- Nearly every day
  1. Feeling down, depressed, or hopeless?
- Not at all
- Several days
- More than half the days
- Nearly every day
  1. Trouble falling or staying asleep, or sleeping too much?
- Not at all
- Several days
- More than half the days
- Nearly every day
  1. Feeling tired or having little energy?
- Not at all
- Several days
- More than half the days
- Nearly every day
  1. Poor appetite or overeating?
- Not at all
- Several days
- More than half the days
- Nearly every day
  1. Feeling bad about yourself or that you are a failure or have let yourself or your family down?
- Not at all
- Several days
- More than half the days
- Nearly every day
  1. Trouble concentrating on things, such as reading the newspaper or watching television?
- Not at all
- Several days
- More than half the days
- Nearly every day
  1. Moving or speaking so slowly that other people could have noticed? Or so fidgety or restless that you have been moving a lot more than usual?
- Not at all
- Several days
- More than half the days
- Nearly every day
  1. Thoughts that you would be better off dead, or thoughts of hurting yourself in some way?
- Not at all
- Several days
- More than half the days
- Nearly every day

**ROME IV DIAGNOSTIC QUESTIONNAIRE FOR ADULTS IRRITABLE BOWEL SYNDROME MODULE**

7.1. In the last 3 months, how often did you have pain anywhere in your abdomen?

- Less than one day a month
- One day a month
- Two to three days a month
- Once a week
- Two to three days a week
- Most days
- Every day
- Multiple times per day or all the time
- Never

7.2. How often did this pain in your abdomen happen close in time to a bowel movement -- just before, during, or soon after? (Percent of times with pain)

- Never 0%
- 10%
- 20%
- 30%
- 40%
- 50%
- 60%
- 70%
- 80%
- 90%
- Always 100%

7.3. How often did your stools become either softer than usual or harder than usual when you had this pain? (Percent of times with pain)

- Never 0%
- 10%
- 20%
- 30%
- 40%
- 50%
- 60%
- 70%
- 80%
- 90%
- Always 100%

7.4. How often did your stools become either more frequent than usual or less frequent than usual when you had this pain? (Percent of times with pain)

- Never 0%
- 10%
- 20%
- 30%
- 40%
- 50%
- 60%
- 70%
- 80%
- 90%
- Always 100%

7.5 Has it been 6 months or longer since you started having this pain?

- Yes
- No

7.6. In the last 3 months, when you had abnormal stools, what were they usually like?


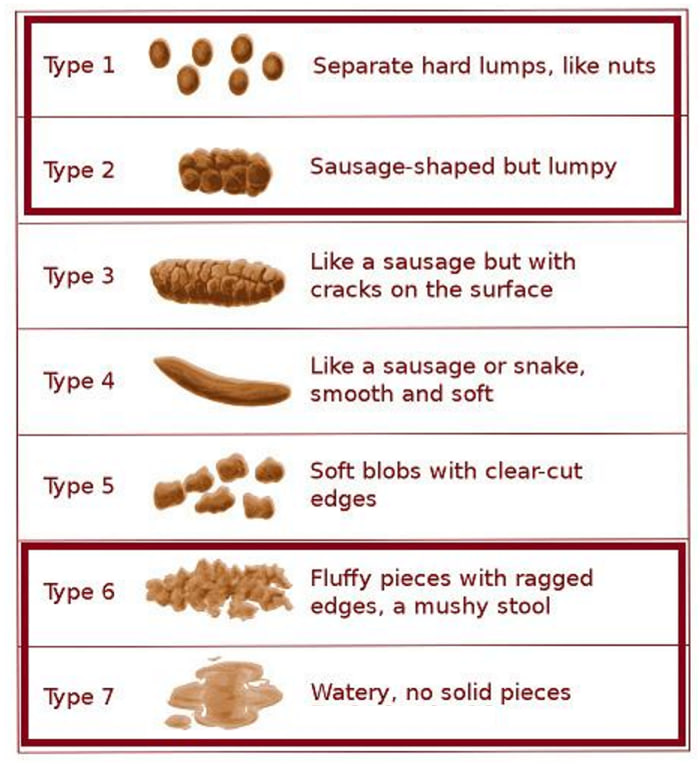


- Usually constipation (like Type 1 or 2 in the picture)
- Usually diarrhea (like Type 6 or 7)
- Both diarrhea and constipation - that is, more than 1/4 of all the abnormal bowel movement were diarrheas and more than 1/4 were constipation
- Not applicable, because I never or rarely had abnormal bowel movements
